# Are acceptance and mindfulness‐based interventions ‘value for money’? Evidence from a systematic literature review

**DOI:** 10.1111/bjc.12208

**Published:** 2018-11-29

**Authors:** Rui Duarte, Annette Lloyd, Eleanor Kotas, Lazaros Andronis, Ross White

**Affiliations:** ^1^ Liverpool Reviews and Implementation Group University of Liverpool UK; ^2^ Psychology Services NHS Greater Glasgow and Clyde UK; ^3^ Populations, Evidence and Technologies Group Division of Health Sciences University of Warwick Coventry UK; ^4^ Division of Clinical Trials University of Warwick Coventry UK; ^5^ School of Psychology University of Liverpool UK

**Keywords:** acceptance and mindfulness‐based interventions, cost‐effectiveness, economic evaluations, mental health, mindfulness, systematic review

## Abstract

**Objectives:**

Acceptance and mindfulness‐based interventions (A/MBIs) are recommended for people with mental health conditions. Although there is a growing evidence base supporting the effectiveness of different A/MBIs for mental health conditions, the economic case for these interventions has not been fully explored. The aim of this systematic review was to identify and appraise all available economic evidence of A/MBIs for the management of mental health conditions.

**Methods:**

Eight electronic bibliographic databases (MEDLINE, MEDLINE In‐Process & Other Non‐Indexed Citations, EMBASE, Web of Science, NHS Economic Evaluation Database (EED), Database of Abstracts of Reviews of Effects (DARE), Health Technology Assessment (HTA) database, and EconLit) were searched for relevant economic evaluations published from each database's inception date until November 2017. Study selection, quality assessment, and data extraction were carried out according to published guidelines.

**Results:**

Ten relevant economic evaluations presented in 11 papers were identified. Seven of the included studies were full economic evaluations (i.e., costs and effects assessed), and three studies were partial economic evaluations (i.e., only costs were considered in the analysis). The A/MBIs that had been subjected to economic evaluation were acceptance and commitment therapy (ACT), dialectical behaviour therapy (DBT), mindfulness‐based cognitive therapy (MBCT), and mindfulness‐based stress reduction (MBSR). In terms of clinical presentations, the evaluation of cost‐effectiveness of A/MBIs has been more focused on depression and emotional unstable personality disorder with three and four economic evaluations, respectively. Three out of seven full economic evaluations observed that A/MBIs were cost‐effective for the management of mental health conditions. Nevertheless, the heterogeneity of included populations, interventions, and economic evaluation study types limits the extent to which firm conclusions can currently be made.

**Conclusion:**

This first substantive review of economic evaluations of A/MBIs indicates that more research is needed before firm conclusions can be reached on the cost‐effectiveness of A/MBIs for mental health conditions.

**Practitioner points:**

- The findings of the review provide information that may be relevant to mental health service commissioners and decision‐makers as all economic evidence available on acceptance and mindfulness‐based interventions for mental health conditions is summarized.
- Evidence relating to the cost‐effectiveness and cost‐saving potential of acceptance and mindfulness‐based interventions is focused mainly on depression and emotional unstable personality disorder to date.
- Heterogeneity in the specific forms of acceptance and mindfulness‐based interventions may limit generalizability of the findings.
- The number of health economic evaluations relating to acceptance and mindfulness‐based interventions remains relatively small. Further research in this area is required.

## Background

Mindfulness‐based interventions (MBIs) have developed over the last 30 years with the intention of averting the negative psychological impacts that arise from a range of medical and psychological disorders. The origins of mindfulness can however be traced back at least 2,500 years to the emergence of Buddhist traditions (Kabat‐Zinn, 2003). The term ‘mindfulness’ has been derived from the Pali word ‘sati’, which derives from the verb ‘Sarati’, which translates ‘to remember’. The development of MBIs has been likened to a process whereby ‘Buddhist meditation has been lifted from its traditional setting in Buddhist doctrine and faith and transplanted in a secularised culture bent on pragmatic results’ (Bodhi, 2011, p. 35). A range of definitions of ‘mindfulness’ have been proposed (Anālayo, 2016). However, a widely cited description suggests that mindfulness involves ‘paying attention in a particular way: on purpose, in the present moment, and non‐judgmentally’ (Kabat‐Zinn, 1994, p. 4). Reflecting on the clinical application of mindfulness, an operational definition describing a two‐component model of a mindfulness intervention has been proposed (Bishop *et al*., 2004). The first component, self‐regulation of attention, focuses on building a participant's capability of feeling fully present in the moment over a prolonged period of time. The second component is an orientation to experience, which facilitates a participant to engage in an active process of acceptance, whereby all emotional and physical symptoms of distress are seen as relevant and to be observed, with the ultimate aim of reducing their impact, due to the subjective change in their meaning.

Mindfulness‐based stress reduction (MBSR) and mindfulness‐based cognitive therapy (MBCT) are commonly used mindfulness interventions. An overview of systematic review and meta‐analysis of randomized control trials (RCTs) found that MBSR and MBCT significantly improved depressive symptoms, anxiety, stress, and physical functioning for patients with cancer, chronic pain, cardiovascular disease, somatic diseases, and depression when compared to waiting list control and to treatment as usual (Gotink *et al*., 2015). Another meta‐analysis which reviewed a range of studies that included these conditions, and also hyperactive disorder, personality disorders, multiple sclerosis, and irritable bowel syndrome, suggested that MBCT is more effective in treating psychological disorders than medical or physical conditions, and is moderately effective in pre–post comparisons, waiting list controls, and comparisons with other active treatments (Khoury *et al*., 2013). Whilst the importance of mindfulness practice is explicitly centred within the practice of MBSR and MBCT, other forms of interventions incorporate mindfulness‐informed acceptance strategies as a key component in a wider package of care. Examples of such interventions include dialectical behavioural therapy (DBT) (Linehan, 2014) and acceptance and commitment therapy (ACT) (Hayes, Pistorello, & Levin, 2012). A meta‐analysis indicated that DBT was effective in reducing self‐destructive behaviours and enhancing the extent to which participants adhered to treatment (Panos, Jackson, Hasan, & Panos, 2014). Similarly, a meta‐analysis of ACT studies concluded that ACT offers promise to reduce distress associated with a range of physical and mental health difficulties (A‐tjak *et al*., 2015). In the research literature acceptance‐ and mindfulness‐based interventions (A/MBIs) are considered together in systematic reviews and meta‐analyses (e.g., Cavanagh, Strauss, Forder, & Jones, 2014; Veehof, Trompetter, Bohlmeijer, & Schreurs, 2016; Vøllestad, Nielsen, & Nielsen, 2012). As with other forms of complex intervention, A/MBIs are complex psycho‐social treatment packages with interrelated components that have a synergizing effect (Demarzo *et al*., 2015).

The most established evidence base for the effectiveness of A/MBIs is centred on patients with depression. Several studies have found MBCT to be effective for recurrent depression compared with usual care and maintenance antidepressants (Kuyken *et al*., 2008, 2016; Ma & Teasdale, 2004; Piet & Hougaard, 2011; Teasdale *et al*., 2000). MBCT as an approach to prevent depressive relapse among patients with three prior episodes of depression is recommended in the United Kingdom (NICE 2009). However, an assessment of the uptake of MBCT in the United Kingdom showed that despite the available evidence and NICE guidance, a relatively low number of mental health services had systematically implemented the recommendation (Crane & Kuyken, 2013). These findings suggest that the role of MBCT in the management of depression has not yet been fully implemented in routine practice.

To support the implementation of A/MBIs, economic evaluations are required to provide a means by which to assess both the costs and consequences of an intervention relative to an alternative course of action (Drummond, Sculpher, Torrance, O'Brien, & Stoddart, 2005). Whilst there are clear insights into the effectiveness of A/MBIs, the evidence base on the cost‐effectiveness of such interventions is sparse (Edwards, Bryning, & Crane, 2015). Such evidence is important in order to ensure that scarce resources are committed to interventions that represent value for money. This systematic review aimed to investigate the cost‐effectiveness of A/MBIs for the treatment of mental health disorders. To do so, this work sets out to (1) consider what type(s) of A/MBIs have been subject to economic evaluation, (2) discern which mental health conditions these interventions have been focussed on treating, (3) evaluate the methodological limitations and strengths of the identified studies, (4) identify knowledge gaps that exist relating to the cost‐effectiveness of A/MBIs, (5) determine whether A/MBIs represent a cost‐effective use of resources for different mental health disorders, and (6) appraise the implications of the review findings for policy, research, and service delivery.

## Methods

This systematic review follows the Preferred Reporting Items for Systematic Reviews and Meta‐Analyses (PRISMA) reporting guidelines (Moher, Liberati, Tetzlaff, & Altman, 2009) and the good practice recommendations for narrative summary of health economic studies outlined in the Cochrane Handbook for Systematic Reviews (Shemilt *et al*., 2011).

### Study identification

Systematic searches were conducted to identify relevant economic evaluations of A/MBIs for the management of mental health conditions as described in the DSM‐5 (American Psychiatric Association 2013). The searches were carried out using the following electronic databases: MEDLINE, MEDLINE In‐Process & Other Non‐Indexed Citations, EMBASE, Web of Science, NHS Economic Evaluation Database (EED), Database of Abstracts of Reviews of Effects (DARE), Health Technology Assessment (HTA) database, and EconLit. The reference lists of relevant systematic reviews and eligible studies were hand‐searched to identify additional potentially relevant studies.

The search strategies were based on terminology linked to A/MBIs and mental health disorders. A search filter designed by NHS EED to identify economic evaluations was applied (Centre for Reviews and Dissemination 2017). No language restriction was applied in the searches. Details of the search strategy for MEDLINE are presented in see Appendix S1. All electronic databases were searched on 22 November 2017. The results of the search strategy were uploaded to and managed using Endnote X8 software.

### Study selection

Full economic evaluations (i.e., cost‐effectiveness analyses, cost‐utility analyses, cost‐benefit analyses, cost‐consequence analyses, or cost‐minimization analyses) and partial economic evaluations (i.e., cost analyses or cost‐description studies) of A/MBIs for participants with mental health disorders as described in the DSM‐5 (American Psychiatric Association 2013) were considered for inclusion in this systematic review. To identify and define A/MBIs, the review drew upon Baer's (2003) empirical review of mindfulness training as a clinical intervention and definition of the key operational characteristics of A/MBIs as proposed by Bishop *et al*. (2004). The following A/MBIs were identified: MBCT, MBSR, DBT, ACT, mindfulness‐based relapse prevention (MBRP), and other mindfulness meditation and mindfulness training.

Two reviewers independently assessed all records based on their title and abstract according to the inclusion criteria (Table 1). Publications that met the inclusion criteria, as well as articles for which an exclusion or inclusion decision could not be made based on their title and abstract alone, were retrieved and judged on the basis of their full text. Disagreements between reviewers were resolved through discussion, and, if necessary, through consulting a third reviewer.

**Table 1:** Inclusion and exclusion criteria

Inclusion criteria (if all of the following met)	Exclusion criteria (if any of the following met)
1. Intervention was an A/MBI	4. Design/protocol paper, methodological paper, (systematic) review, meta‐analysis, commentaries/editorial
2. Intervention was targeted at adults with mental health disorders as described in the DSM‐5	5. Insufficient information (e.g., study only available as a conference proceeding/abstract)
3. Full or partial economic evaluation	

Note

A/MBI = acceptance‐ and mindfulness‐based intervention; DSM = Diagnostic and Statistical Manual of Mental Disorders.

### Data extraction

Relevant data from the full‐text papers eligible for inclusion were extracted using a data extraction form designed for the purposes of this study. Such data included bibliographic information (author[s] and year of publication); general information (country, condition, intervention, and comparator[s]); methodological characteristics (type of economic evaluation, perspective, time horizon, discount rate, key cost categories, year of valuation, and key outcomes), and main findings. One author extracted the data, and a second author checked the data for accuracy. Disagreements were resolved through discussion between reviewers and, when necessary, by seeking the opinion of a third reviewer. A narrative synthesis was used to interpret, summarize, and present the information provided in the selected articles.

### Quality assessment

The methodological quality of the economic evaluations identified was assessed using the Consensus on Health Economic Criteria checklist (CHEC‐list) (Evers, Goossens, de Vet, van Tulder, & Ament, 2005), which is recommended for appraising the methodological quality of economic evaluations (Higgins & Green, 2011). The CHEC‐list comprises 19 questions to be answered as ‘yes’, ‘no’, ‘unclear’, or ‘not applicable’. Negative answers to the CHEC‐list do not necessarily indicate poor practice or result in bias (i.e., it may relate to absence of information rather than methodological issues). Quality assessment was carried out by one reviewer and checked for agreement by a second reviewer. Disagreements were resolved through discussion between reviewers and, when necessary, by seeking the opinion of a third reviewer.

## Results

**Figure 1:**
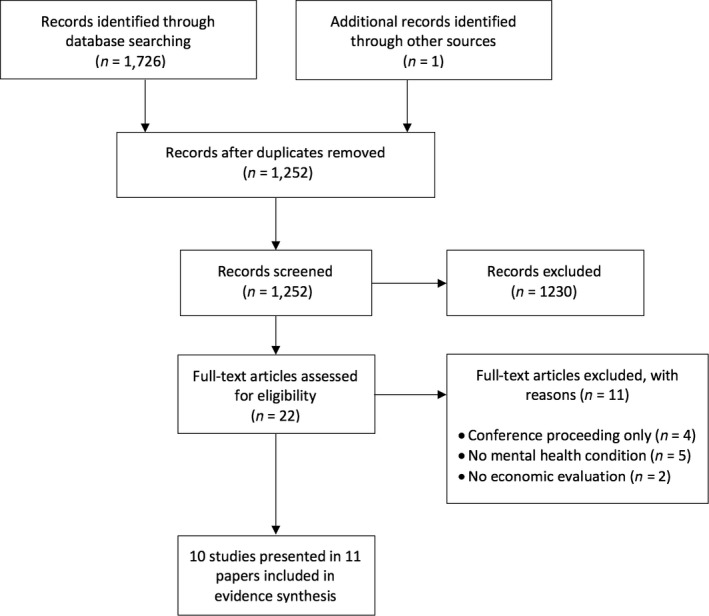
PRISMA flow chart detailing the study selection process.

A total of 1,727 records were retrieved, of which 1,726 were identified through searches of electronic databases and one was identified through supplementary searches, that is hand‐search of reference lists (Figure 1). Following removal of 475 duplicate records, the title and abstract of 1,252 unique articles were screened. Title and abstract screening of these articles led to the exclusion of 1230 records. The 22 potentially relevant studies were retrieved, and their full text was assessed for eligibility. Consideration of these studies against the selection criteria led to the exclusion of a further 11 references (Bota, Hazen, Tieu, & Novac, 2016; Fjorback *et al*., 2012, 2013; Holmes *et al*., 2007; Lengacher *et al*., 2015; Luciano *et al*., 2017; McDaid & Park, 2012; Murphy & Bourke, 2014; Pots *et al*., 2016; Prioli *et al*., 2017; Van Roijen, Sinnaeve, Bouwmans, & Van Den Bosch, 2015). The remaining 10 studies presented in 11 articles formed the final set of reviewed evidence (Amner, 2012; Finnes *et al*., 2017; Knight, Bean, Wilton, & Lin, 2015; Kuyken, *et al*., 2008; Kuyken, *et al*., 2015a,b; Pasieczny & Connor, 2011; Priebe *et al*., 2012; Shawyer, Enticott, Ozmen, Inder, & Meadows, 2016; van Ravesteijn *et al*., 2013; Wagner *et al*., 2014).

### Overview of included studies

The included studies consider the health economic impact of A/MBIs across a range of mental health conditions. Characteristics of the 10 studies are presented in Table 2. Seven of the studies were conducted in Europe (four in the United Kingdom, and one in each of Sweden, the Netherlands, and Germany), two in Australia, and one in Canada. All of the studies were published after 2008. Seven of the included studies investigated the cost‐effectiveness of A/MBIs being delivered for emotional unstable personality disorder (EUPD) and depression (three or more previous major depressive episodes), whilst three studies explored different conditions (i.e., mental health disorders and medically unexplainable symptoms).

**Table 2:** General characteristics of the economic evaluations included

Author (year) Country	Condition	Intervention	Comparator(s)
Amner (2012) UK	EUPD symptoms and characteristics such as emotional dysregulation and recent self‐harming behaviours (during the previous 12 months) (*n* = 21)	DBT weekly sessions over 1 year comprising a two and a half hourly educational training group focusing on the acquisition of psycho‐social skills, combined with individual hour‐long sessions with a suitably trained therapist	UC (treatment received in the year prior to engagement in DBT)
Finnes *et al*. (2017) Sweden	Mental health disorders including anxiety disorder, depression, reaction to severe stress, or adjustment disorder (*n* = 352)	ACT comprising six individual 60‐min sessions	ACT+WDI (intervention + three meetings involving the participant and his or her supervisor at work); WDI alone (three meetings involving the participant and his or her supervisor at work); UC (any intervention or consultation as offered by the primary care centre or other care facility; typically CBT and/or pharmacological treatments but also physical therapy and counselling
Knight *et al*. (2015) Canada	Mental health issues (described as people referred by their physician for a variety of mental health issues) (*n* = 1,730)	MBSR, 10‐weeks programme consisted of nine weekly 3‐hr group classes, daily homework assignments, and one 7‐hr class	UC (health service use before participation in the MBSR programme)
Kuyken *et al*. (2008) UK	Depression (three or more previous episodes of depression and on maintenance antidepressant medication for at least the previous 6 months) (*n* = 123)	MBCT delivered in primary care settings with groups of 9–15 patients, 2‐hr sessions over 8 consecutive weeks, followed by four follow‐up sessions in the following year, and antidepressant tapering/discontinuation support (discussion with physicians after 4–5 weeks of the MBCT groups)	Maintenance antidepressant treatment monitored and treated by their physicians in primary care settings. During the maintenance phase, physicians were asked to manage antidepressant treatment in line with standard clinical practice and the BNF
Kuyken, Hayes, Barrett, Byng, Dalgleish, Kessler, Lewis, Watkins, Brejcha, *et al*. (2015a) UK	Depression (three or more previous major depressive episodes, recurrent major depressive disorder in full or partial remission, and on a therapeutic dose of maintenance antidepressant) (*n* = 424)	MBCT consisting of eight 2.25‐hr group sessions, normally over consecutive weeks, with four refresher sessions offered roughly every 3 months for the following year, and antidepressant tapering/discontinuation support	Maintenance antidepressant treatment, patients received support from their GPs to maintain a therapeutic level of antidepressant medication in line with BNF and NICE guidelines
Pasieczny and Connor (2011) Australia	EUPD; BPD according to DSM‐IV‐TR criteria; all patients had at least one additional DSM axis 1 comorbid diagnosis, most commonly substance use disorders (51%), depressive disorders (77%), bipolar affective disorder (6%), PTSD (23%), other anxiety disorders (50%), and schizophrenia (4%) (*n* = 92)	DBT taking place over 6 months and consisting of weekly individual psychotherapy (1 h), weekly group skills training (2 h), access to phone coaching between sessions, and therapist attendance at a weekly DBT consultation meeting (1.5 h)	UC consisting of engagement, ongoing assessment, planning, linking with community resources, consultation with carers, assistance expanding social networks, collaboration with medical staff, advocacy, individual counselling, living skills training, psycho‐education, and crisis management
Priebe *et al*. (2012) UK	EUPD (5 days or more with self‐harm in the year prior to treatment, and a diagnosis of at least one personality disorder; the majority of patients (91%) have a diagnosis of EUPD) (*n* = 80)	DBT consisting of weekly hour‐long individual therapy sessions, a weekly 2‐hr skills training group, and out‐of‐hours skills coaching over the telephone as needed during 12 months	UC which may have included treatment from psychotherapists, psychiatrists, community mental health teams, counsellors, GPs, or user‐run support groups
Shawyer *et al*. (2016) Australia	Depression (three or more previous major depressive episodes, recurrent major depressive disorder) (*n* = 204)	MBCT consisting of eight 2‐hr group training sessions delivered weekly and 3‐monthly optional ‘booster sessions’; and depressive relapse active monitoring consisting of monthly supported self‐monitoring using the Patient Health Questionnaire‐2 and ‐9	Depressive relapse active monitoring alone which involved monthly supported self‐monitoring using the Patient Health Questionnaire‐2 and Patient Health Questionnaire‐9
van Ravesteijn *et al*. (2013) Netherlands	MUS (patients fulfilled the DSM‐IV criteria of an undifferentiated somatoform disorder) (*n* = 125)	MBCT consisting of eight 2.5‐hr group sessions from experienced mindfulness trainers. Participants were instructed to practice at home 6 days a week for approximately 45 min a day	EUC provided by their GP and other health care professionals. It is considered EUC as all patients received a psychiatric interview and the GP was explicitly informed about the psychiatric diagnoses resulting from the interview
Wagner *et al*. (2014) Germany	EUPD (patients included if they met at least five BPD criteria according to DSM‐IV‐TR) (*n* = 47)	DBT with all patients receiving weekly individual therapy (50 Min), 89.4% of patients participated in a weekly skills training group (120 Min), telephone coaching was offered as needed and about 85% of the therapists participated in a weekly or biweekly consultation team (50 min). After the DBT treatment year, there was an option to continue individual therapy if indicated.	UC concerned the treatment received in the year before DBT

Note

ACT = acceptance and commitment therapy; BNF = British National Formulary; BPD = borderline personality disorder; CBT = cognitive behavioural therapy; DBT = dialectical behaviour therapy; DSM = Diagnostic and Statistical Manual of Mental Disorders; EUC = enhanced usual care; EUPD = emotionally unstable personality disorder; GPs = general practitioners; MBCT = mindfulness‐based cognitive therapy; MBSR = mindfulness‐based stress reduction; MUS = medically unexplainable symptoms; NICE = National Institute for Health and Care Excellence; PTSD = post‐traumatic stress disorder; UC = usual care; WDI = workplace dialogue intervention.

Five of the 10 studies explicitly described the intervention as being ‘mindfulness‐based’. Four of these five studies assessed MBCT, and one study evaluated MBSR. A combination of MBCT with antidepressant tapering/discontinuation support or depressive relapse active monitoring was employed in the three studies evaluating MBCT for major depressive disorder. The remaining five studies integrated mindfulness as a key component with other psychotherapy approaches. Four of these five studies evaluated DBT, and one study investigated ACT. All of the 10 studies indicated that mindfulness practice was a fundamental aspect of the intervention provided.

Seven studies stated that the A/MBIs were compared to a form of usual care, including enhanced usual care (defined as such because all participants received a psychiatric interview and the GP was explicitly informed about the psychiatric diagnoses resulting from the interview). Two other studies can be considered to use usual care as the comparator to the A/MBIs since participants in the control group just continued to receive antidepressant treatment. One study evaluated ACT versus ACT combined with another intervention (i.e., workplace dialogue intervention [WDI]), WDI alone, and usual care.

### Assessment of methodological quality

Overall, the majority of the studies appeared to meet the majority of the quality checklist criteria as detailed in Evers *et al*. (2005). The economic evaluations assessing A/MBIs for depression (Kuyken, *et al*., 2008; Kuyken, Hayes, Barrett, Byng, Dalgleish, Kessler, Lewis, Watkins, Brejcha, *et al*., 2015a; Kuyken, Hayes, Barrett, Byng, Dalgleish, Kessler, Lewis, Watkins, Morant, *et al*., 2015b; Shawyer *et al*., 2016) met most of the criteria. The three studies meeting the least number of criteria were Amner (2012), Knight *et al*. (2015), and Wagner *et al*. (2014). It should be noted that the studies meeting the least number of the criteria (Amner, 2012; Knight *et al*., 2015; Wagner *et al*., 2014) were partial economic evaluations with outcome data derived from either a before and after study design (Amner, 2012; Wagner *et al*., 2014) or a matched‐control database study (Knight *et al*., 2015). The assessment of the methodological quality of the 10 economic evaluations is presented in Appendix S2.

### Characteristics of the included economic evaluations

A summary of the design characteristics of the 10 economic evaluations included is presented in Table 3. Seven of the 10 included studies were full economic evaluations, with the remaining three representing partial economic evaluations. Of the seven full economic evaluations, two were cost‐effectiveness analyses (Kuyken *et al*., 2008; Priebe *et al*., 2012), two were cost‐utility analyses (Finnes *et al*., 2017; van Ravesteijn *et al*., 2013), one was a cost‐consequence analysis (Pasieczny & Connor, 2011), and two economic evaluations included both a cost‐effectiveness and a cost‐utility analysis (Kuyken, Hayes, Barrett, Byng, Dalgleish, Kessler, Lewis, Watkins, Brejcha, *et al*., 2015a; Kuyken, Hayes, Barrett, Byng, Dalgleish, Kessler, Lewis, Watkins, Morant, *et al*., 2015b; Shawyer *et al*., 2016). The three partial economic evaluations were cost comparisons (Amner, 2012; Knight *et al*., 2015; Wagner *et al*., 2014). No economic models evaluating the long‐term cost‐effectiveness of A/MBIs were identified. The resource use and effectiveness data that informed the economic evaluations were derived from single studies.

**Table 3:** Methods employed in the economic evaluations included

Author (year)	Perspective	Type of economic evaluation (analytic method employed)	Time horizon (discounting and rate)	Main cost categories and year of valuation	Measure of benefit (instrument)
Amner (2012)	Health care system	CC (before–after study)	36 months (discounting not performed)	Treatment‐ and hospital care‐related costs 1‐year before, during, and 1‐year after DBT Valuation year: 2010	N/A
Finnes *et al*. (2017)	Health care system and societal	CUA (trial‐based economic evaluation)	12 months (discounting N/A)	Intervention costs and health care costs impacted by the interventions and sickness benefits Valuation year: 2015	QALY (EQ‐5D‐3L)
Knight *et al*. (2015)	Third‐party payer	CC (matched‐control database study)	48 months (discounting not performed)	Claims submitted by physicians, emergency department visits, and inpatient discharges Valuation year not provided	N/A
Kuyken *et al*. (2008)	Health care system and societal	CEA (trial‐based economic evaluation)	15 months (discounting not performed)	Hospital and community health and social services, plus productivity losses resulting from time off work due to illness Valuation year: 2005–2006	Relapse prevented and depression‐free day
Kuyken, *et al*. (2015a,b)	Health care system and societal	CEA, CUA (trial‐based economic evaluation)	24 months (3.5% per year for costs and outcomes)	Hospital and community health and social services, plus productivity losses Valuation year: 2011–2012	Relapse/recurrence prevented QALY (EQ‐5D‐3L)
Pasieczny and Connor (2011)	Health care system	CCA (trial‐based economic evaluation)	6 months (discounting N/A)	Treatment‐ and hospital care‐related costs Valuation year not provided	Frequency of suicide attempts and decrease of NSSI
Priebe *et al*. (2012)	Societal	CEA (trial‐based economic evaluation)	12 months (discounting N/A)	Hospital and social services and lost work days Valuation year not provided	Decrease in self‐harm rates
Shawyer *et al*. (2016)	Health care system and societal	CEA, CUA (trial‐based economic evaluation)	24 months (3% per year for costs)	Hospital and community health and social service contacts. Productivity losses resulting from days off work Valuation year: 2009	Depression‐free day DALY
van Ravesteijn *et al*. (2013)	Health care system and societal	CUA (trial‐based economic evaluation)	12 months (discounting N/A)	Hospital and community health and social services, plus productivity losses resulting from work absence Valuation year: 2010	QALY (SF‐6D)
Wagner *et al*. (2014)	Societal	CC (before–after study)	36 months (discounting not performed)	Medical and non‐medical resource consumption, informal care (including significant others volunteering to take over domestic tasks without payment) and productivity loss Valuation year: 2010	N/A

Note

CC = cost comparison; CCA = cost‐consequence analysis; CEA = cost‐effectiveness analysis; CUA = cost‐utility analysis; DALY = disability‐adjusted life year; N/A = not applicable; QALY = quality‐adjusted life year; NSSI = non‐suicidal self‐injury.

Seven of the 10 studies adopted a societal perspective, in which all costs and benefits associated with the interventions in question were taken into account, irrespective of what entity they accrued to (Finnes *et al*., 2017; Kuyken, *et al*., 2008; Kuyken, Hayes, Barrett, Byng, Dalgleish, Kessler, Lewis, Watkins, Brejcha, *et al*., 2015a; Kuyken, Hayes, Barrett, Byng, Dalgleish, Kessler, Lewis, Watkins, Morant, *et al*., 2015b; Priebe *et al*., 2012; Shawyer *et al*., 2016; van Ravesteijn *et al*., 2013; Wagner *et al*., 2014). Two studies carried out their analysis from a health and social care perspective (Amner, 2012; Pasieczny & Connor, 2011), whilst one study adopted a third‐party payer perspective (Knight *et al*., 2015). Five of the seven studies conducted from a societal perspective also presented results specific to a health and social care perspective (Finnes *et al*., 2017; Kuyken, *et al*., 2008; Kuyken, Hayes, Barrett, Byng, Dalgleish, Kessler, Lewis, Watkins, Brejcha, *et al*., 2015a; Kuyken, Hayes, Barrett, Byng, Dalgleish, Kessler, Lewis, Watkins, Morant, *et al*., 2015b; Shawyer *et al*., 2016; van Ravesteijn *et al*., 2013).

Six of the 10 studies had a study time horizon > 12 months, and two of the studies (Kuyken, Hayes, Barrett, Byng, Dalgleish, Kessler, Lewis, Watkins, Brejcha, *et al*., 2015a; Kuyken, Hayes, Barrett, Byng, Dalgleish, Kessler, Lewis, Watkins, Morant, *et al*., 2015b; Shawyer *et al*., 2016) had specifically discounted the value of the costs accruing in the future at a rate of 3.5% and 3.0% per annum, respectively. The study by Kuyken, Hayes, Barrett, Byng, Dalgleish, Kessler, Lewis, Watkins, Brejcha, *et al*. (2015a) was the only study to have reported discounting the effects, as well as the costs, both at a rate of 3.5% per annum.

Sensitivity analyses feature in four of the full economic evaluations as a way of dealing with uncertainty associated with a range of parameters (Finnes *et al*., 2017; Kuyken, Hayes, Barrett, Byng, Dalgleish, Kessler, Lewis, Watkins, Brejcha, *et al*., 2015a; Kuyken, Hayes, Barrett, Byng, Dalgleish, Kessler, Lewis, Watkins, Morant, *et al*., 2015b; Shawyer *et al*., 2016; van Ravesteijn *et al*., 2013). This was also the case in two of the three partial economic evaluations (Amner, 2012; Wagner *et al*., 2014).

### Results of the included economic evaluations

The results of the economic evaluations are summarized in Table 4 and discussed below according to the conditions addressed by the studies.

**Table 4:** Findings of the economic evaluations included

Author (year)	Main findings
Amner (2012)	DBT results in a cost‐saving of £36,551 between Years 1 (prior to DBT) and 3 (after DBT) as a result of reduced health service use Mean cost saving per participant overall was £1,741 (median: £1,059, range: £46,264–£55,461)
Finnes *et al*. (2017)	*Health care perspective:* ACT results in an ICER of $33,579 per additional QALY gained compared with the ‘null comparator’ ACT+WDI results in an ICER of $158,500 per additional QALY gained compared with ACT There is a 60% probability of ACT being more cost‐effective than doing nothing when WTP per unit health gain is $6650, which increases to 75% if WTP is $14,000. For ACT+WDI compared with ACT, the probability of cost‐effectiveness reaches a maximum of 50% at a WTP of $11,000 per unit health gain Societal perspective: ICER for ACT was $88,122, followed by ACT+WDI with an ICER of $653,500 per QALY gained WDI and UC were dominated in both health care system and societal perspectives
Knight *et al*. (2015)	The mean OHIP cost for the cases dropped by $244 to $279, whilst the mean costs for the controls increased between $3 and $18
Kuyken *et al*. (2008)	*Health care system perspective:* ICER $439 per relapse/recurrence prevented; ICER $23 per depression‐free day *Societal perspective:* ICER $962 per relapse/recurrence prevented; ICER $50 per depression‐free day The probability of MBCT being the more cost‐effective of the two options increases as the WTP for preventing an additional relapse/recurrence increases, suggesting that MBCT has a higher probability of being more cost‐effective than has maintenance antidepressant treatment for WTP levels of approximately $1,000 and above for preventing an additional relapse/recurrence
Kuyken, *et al*. (2015a)	*Health care system perspective:* ICER £4955 per unit reduction in the % of patients who relapse *Societal perspective:* ICER £17,930 per unit reduction in the % of patients who relapse The probability of MBCT being more cost‐effective to prevent relapses than maintenance antidepressant treatment does not rise above 43% In terms of QALYs, costs were higher in the MBCT group and outcomes slightly worse, suggesting that MBCT was dominated by maintenance antidepressant treatment The probability that MBCT is more cost‐effective than maintenance antidepressant treatment does not rise above 52%
Pasieczny and Connor (2011)	DBT resulted in a saving of $5927 per patient when compared to UC Patients in the DBT group demonstrated a greater multivariate improvement across suicide attempts and NSSI (Hotelling's *T* = 25.13, *F*(2, 78) = 25.14, *p* < .001)
Priebe *et al*. (2012)	£36 to achieve a one percentage point reduction in the incidence of self‐harm as a result of using DBT
Shawyer *et al*. (2016)	*Health care system perspective:* saving of AUS $83 to avert a day of major depression in one person by intervening with MBCT MBCT produced a saving of AUS $87,313 per person per year by averting a DALY *Societal perspective:* saving of AUS $156 to avert a day of major depression in one person by intervening with MBCT MBCT produced a saving of AUS $164,768 per person per year by averting a DALY The probability of MBCT being cost‐effective when the decision‐maker is unwilling to pay anything additional for an extra point increase in DALY is 81% from the mental health budget perspective, 62% at primary care level, and 83% at specialist care level
van Ravesteijn *et al*. (2013)	*Health care system perspective:* ICER of €66,450 per QALY gained *Societal perspective:* ICER of €56,637 per QALY gained The probability that MBCT is cost‐effective is 55% assuming a WTP of €80,000 for a QALY gain
Wagner *et al*. (2014)	Total mean annual EUPD‐related societal cost‐of‐illness was €28,026 during pre‐treatment, €18,758 during the DBT treatment year and €14,750 during the follow‐up year

Note

DALY = disability‐adjusted life year; DBT = dialectical behaviour therapy; EUPD = emotionally unstable personality disorder; ICER = incremental cost‐effectiveness ratio; MBCT = mindfulness‐based cognitive therapy; NSSI = non‐suicidal self‐injury; OHIP = Ontario Health Insurance Plan; QALY = quality‐adjusted life year; UC = usual care; WTP = willingness to pay.

#### Depression

One of the three studies assessing the impact of MBCT on depression (i.e., three or more previous major depressive episodes) yielded a positive result with regard to cost‐effectiveness, with Shawyer *et al*. (2016) reporting a 83% probability of MBCT being cost‐effective compared to usual care, from a mental health care perspective, whilst Kuyken *et al*. (2008) reporting a 42% probability of MBCT plus antidepressant tapering being cost‐effective based on a society's willingness to pay (WTP) of zero to prevent an additional relapse/recurrence. The authors report that there was a higher probability of cost‐effectiveness, if the WTP threshold to prevent an additional relapse/recurrence was set at a minimum of $1,000. However, Kuyken, Hayes, Barrett, Byng, Dalgleish, Kessler, Lewis, Watkins, Brejcha, *et al*. (2015a), Kuyken, Hayes, Barrett, Byng, Dalgleish, Kessler, Lewis, Watkins, Morant, *et al*. (2015b) study into the cost‐effectiveness of MBCT compared to antidepressant treatment, found the costs in the intervention group to be higher and the effectiveness smaller than in the comparator group (i.e., dominated), and reported the probability of MBCT being cost‐effective as no greater than 43%.

#### Emotional unstable personality disorder

All of the four studies that reviewed the health economic impact of A/MBIs on participants with EUPD assessed DBT as the intervention. The partial evaluation by Amner (2012) found that DBT had a positive impact on cost with an average saving per patient of £1,741 based on the year prior to intervention and the year following DBT. The cost comparison by Wagner *et al*. (2014) observed a reduction in societal cost‐of‐illness in the year after the intervention. However, these studies (Amner, 2012; Wagner *et al*., 2014) were partial economic evaluations and met a lesser number of criteria in the quality assessment than the other studies assessing DBT for EUPD. The remaining two (full) evaluations obtained positive results with Priebe *et al*. (2012) reporting a modest incremental cost of £36 to achieve a one percentage point reduction in the incidence of self‐harm; however, no explicit statement on the probability of cost‐effectiveness was reported. Pasieczny and Connor (2011) adopting a CCA approach reported a positive impact of DBT on both cost and outcomes, with a mean saving of AUS $5,927 per patient over a 6‐month period and mean reduction in suicide attempts of 1.34 per patient, respectively.

#### Remaining conditions

The remaining three studies assessed the economic impact of A/MBIs for medically unexplainable symptoms and mental health disorders without specifying these disorders. The study investigating the impact of MBCT for participants with medically unexplained symptoms reported a 57% probability of cost‐effectiveness against a WTP threshold of EUR 80,000 per quality‐adjusted life year (QALY) (van Ravesteijn *et al*., 2013). ACT for mental health disorders was found to have a probability of being cost‐effective of 75% at a WTP of $14,000 per unit health gain (Finnes *et al*., 2017). An assessment of MBSR also for mental health disorders observed a reduction in mean insurance costs per case treated (Knight *et al*., 2015). However, the study by Knight *et al*. (2015) was a partial economic evaluation and met a lesser number of criteria in the quality assessment than the full economic evaluations.

## Discussion

There is growing evidence supporting the effectiveness of A/MBIs in ameliorating distress associated with a range of mental health conditions. However, to date there have been no published systematic reviews that seek to synthesize and evaluate data relating to the cost‐effectiveness of A/MBIs. The present systematic review investigated whether A/MBIs for the management of mental health conditions represent a cost‐effective use of limited resources across the diverse settings in which the interventions have been applied. As such, this review aimed to investigate the extent to which A/MBIs had been evaluated from a cost‐effectiveness perspective, to examine the types of mental health conditions these interventions had focussed on treating, and to appraise the strengths and limitations of these evaluations from a methodological perspective.

A total of 10 economic evaluations of A/MBIs were included in this review. Not all studies presented an incremental analysis, and those that did present an incremental analysis used different WTP thresholds per QALY gain or improvement in outcomes assessed; therefore, the results from this systematic review need to be interpreted with caution. Three studies (Finnes *et al*., 2017; Shawyer *et al*., 2016; and van Ravesteijn *et al*., 2013) considered A/MBIs to be cost‐effective when compared to the respective comparator treatments. However, van Ravesteijn *et al*. (2013) used a WTP of €80,000 per QALY gained, which is higher than the WTP per QALY gained in other countries. Three partial economic evaluations (Amner, 2012; Knight *et al*., 2015; Wagner *et al*., 2014) with inherent quality limitations observed cost‐savings with the use of A/MBIs. In the study by Kuyken *et al*. (2008), the use of MBCT was considered to be justified but only if the WTP for improvements in the proportion of patients who relapse was $1,000 or above and results were considered inconclusive. One study concluded that MBCT was not cost‐effective (Kuyken, Hayes, Barrett, Byng, Dalgleish, Kessler, Lewis, Watkins, Brejcha, *et al*., 2015a; Kuyken, Hayes, Barrett, Byng, Dalgleish, Kessler, Lewis, Watkins, Morant, *et al*., 2015b), and the remaining two studies (Pasieczny & Connor, 2011; Priebe *et al*., 2012) presented inconclusive results as to the cost‐effectiveness of A/MBIs. In keeping with A/MBI transdiagnostic credentials (where promoting non‐judgemental openness to experience, and not eradicating symptoms, is the key aim), the studies focused on a range of conditions. Nevertheless, the current review did yield three economic evaluations, which focussed on MBCT for major depressive disorder and four studies evaluating the cost‐effectiveness of DBT for EUPD. As such, presenting the findings of this review by condition type provides an opportunity to examine the relative congruency of results, particularly for these two specific conditions.

The three studies assessing the impact of MBCT on patients with major depressive disorder yielded a variable set of cost‐effectiveness results. The study by Kuyken *et al*. (2008) produced an inconclusive result regarding cost‐effectiveness, whilst the more recent study by Kuyken, Hayes, Barrett, Byng, Dalgleish, Kessler, Lewis, Watkins, Brejcha, *et al*. (2015a) reported that the MBCT group resulted in more costs and less effectiveness than maintenance antidepressant treatment group. The findings also indicated either marginal borderline evidence that MBCT reduced the risk of relapse of a depressive episode relative to the comparator treatment (Kuyken *et al*., 2008), or no difference at all (Kuyken, Hayes, Barrett, Byng, Dalgleish, Kessler, Lewis, Watkins, Brejcha, *et al*., 2015a; Kuyken, Hayes, Barrett, Byng, Dalgleish, Kessler, Lewis, Watkins, Morant, *et al*., 2015b).

Nevertheless, the evaluation by Shawyer *et al*. (2016) into MBCT combined with depressive relapse monitoring yielded a strong result with regard to cost‐effectiveness of this integrated approach versus the comparator treatment of depressive relapse monitoring alone. Indeed, participants in the intervention arm of the trial spent less time, on average, in a major depressive state and as result incurred less DALYs relative to the comparator treatment (Shawyer *et al*., 2016). The studies by Kuyken, Hayes, Barrett, Byng, Dalgleish, Kessler, Lewis, Watkins, Brejcha, *et al*. (2015a) and Shawyer *et al*. (2016) are similar in terms of the criteria met in the quality assessment; however, the study by Shawyer *et al*. (2016) did not discount the outcomes as recommended it should be done in economic evaluations with a time horizon longer than 12 months. In summary, the results arising from the three studies fail to provide conclusive evidence that MBCT is a cost‐effective treatment for depression.

The review identified four studies that evaluated the health economic impact of DBT on patients with EUPD. None of these studies provided convincing evidence to support a hypothesis that A/MBIs are a cost‐effective treatment, due in part to economic evaluation design and uncertainty around results. For example, whilst both the studies by Amner (2012) and Wagner *et al*. (2014) reported that the cost of treating participants with EUPD reduced after DBT intervention when compared with treatment prior to DBT, there was no assessment of health outcomes and pre‐intervention data were collected retrospectively. Furthermore, given the small sample sizes, limited scope of cost, and a broad approach to sensitivity analysis, the results can be viewed as uncertain.

Whilst Pasieczny and Connor (2011) did not conduct an incremental analysis of DBT, the study revealed that participants consumed fewer health and social care resources on the DBT arm of the trial and benefited from better health outcomes on the back of reduced incidence of suicide attempts. However, the results relating to cost have a degree of uncertainty, as the mean reduction of AUS $5,927 per patient on the DBT arm related specifically to a 6‐month time horizon, and the authors reported the intervention group to be more costly than the comparator treatment group for those patients who continued to receive the intervention for a further 6 months.

Similarly, the study of Priebe *et al*. (2012) evidenced a reduction in self‐harm in participants receiving DBT intervention arm compared to the treatment as usual arm, and the mean cost per patient of the DBT intervention was higher than the comparator, albeit not statistically significant. The level of uncertainty around the cost impact of the intervention led the authors to conclude that whilst DBT was shown to be effective in reducing the incidence of self‐harm, there was a possibility for higher treatment costs overall. So whilst the incremental cost to achieve a one percentage point reduction in the incidence of self‐harm was relative low, the small sample size and lack of statistical power to yield a statistically significant difference in cost create a level of uncertainty around cost‐effectiveness, which was not explored by the authors.

It is difficult to draw any direct comparisons between the remaining three studies that evaluated the impact of A/MBIs on non‐specific mental health conditions. Nevertheless, the study by van Ravesteijn *et al*. (2013) evaluating MBCT for the treatment of medically unexplained symptoms reported that the probability of cost‐effectiveness was 55% assuming a WTP of €80,000 per QALY gained. Finnes *et al*. (2017) reported a 75% probability of ACT being cost‐effective when compared to doing nothing at a willingness‐to‐pay threshold of $14,000 per QALY gained.

### Methodological considerations

Whilst this review contributes to the knowledge base of what is known about the economic impact of A/MBIs, there are a number of methodological issues to be considered when assessing the internal and external validity of the identified studies. A/MBIs are considered to be psycho‐social interventions targeting the psychological effects of a range of mental health conditions. As such, we contend that it is appropriate to adopt a wide perspective when quantifying all relevant costs, despite NICE guidance (NICE 2013) recommending the adoption of a narrower health and personal social care perspective for the purposes of decision‐making within the context of the NHS. Seven of the papers included in this review adopted a societal perspective, and five of the studies included presented a set of results from both a societal and a health and personal social care perspective. Indeed, it is noteworthy that two of evaluations focusing on MBCT's effect on depression (Kuyken, *et al*., 2008; Kuyken, Hayes, Barrett, Byng, Dalgleish, Kessler, Lewis, Watkins, Brejcha, *et al*., 2015a; Kuyken, Hayes, Barrett, Byng, Dalgleish, Kessler, Lewis, Watkins, Morant, *et al*., 2015b) presented results in this way, thus strengthening their external validity from this perspective.

Closely allied to the issue of perspective is the method by which all relevant costs are sourced and measured. The current review indicated that there was large variability across the studies to how successful this had been executed. For example, Amner (2012), due primarily to data availability issues, only included mental health service resource use. There were, however, a number of studies in the current review that adopted a robust approach to defining all relevant costs. For example, the three studies that focused on the cost‐effectiveness of MBCT for the treatment of depression had a comprehensive set of costs assumed within the analysis (Kuyken, *et al*., 2008; Kuyken, Hayes, Barrett, Byng, Dalgleish, Kessler, Lewis, Watkins, Brejcha, *et al*., 2015a; Kuyken, Hayes, Barrett, Byng, Dalgleish, Kessler, Lewis, Watkins, Morant, *et al*., 2015b; Shawyer *et al*., 2016).

In relation to benefits, the identified studies employed a variety of measures to capture positive changes in mental health outcomes and improved health‐related quality of life, with the latter being translated into QALYs. QALYs offer a generic measure of quality of life (QoL) that can facilitate comparisons across areas and conditions, including mental health problems (Brazier, 2008). There is evidence supporting the use of generic QoL measures to evaluate interventions for common mental health problems (e.g., depression and anxiety), but for other mental health conditions such as schizophrenia, a preference‐based measure focused on the impact of mental health should be considered (Mulhern *et al*., 2014). However, the merits of using QALYs over, arguably, narrower measures of outcomes need to be balanced against the fact that such a generic measure, at least in its current form, may be insensitive to changes in mental health. For example, empirical evidence suggests that psychological well‐being and social interaction are salient elements for people with mental health problems, though such elements are not included in prominent generic measures of QoL, which instead focus on domains such as usual activities, physical mobility, and pain (Chisholm, Healey, & Knapp, 1997). Additional shortcomings of expressing changes in mental health through generic QoL measures relate to the valuation and nature of care required. Taken together, these issues suggest a need for caution when using generic measures for the evaluation of mental health conditions.

It is positive that nine of the ten studies had a study time horizon of 12 months or more, with two of the three studies assessing MBCT for depression having a 24‐month follow‐up. Given the multi‐dimensional nature of an A/MBI, the time horizons adopted in these studies provided a useful base to capture the effect of the A/MBI, congruent with Medical Research Council guidance (Craig *et al*., 2008). With the exception of the Kuyken, Hayes, Barrett, Byng, Dalgleish, Kessler, Lewis, Watkins, Brejcha, *et al*. (2015a) and Shawyer *et al*. (2016) studies, the economic evaluations in the current review were dependent on the results of relatively small trials, which may affect the internal validity of the results. Furthermore, whilst six of the ten studies were reported to have undertaken sensitivity analysis to test various parameter uncertainties, the data and methods used to characterize the level of uncertainty were either not described or not clearly detailed.

### Strengths and limitations of the review

Each part of the review, including study identification, selection, quality assessment, and data extraction, was carried out in line with PRISMA (Moher *et al*., 2009) and Centre for Reviews and Dissemination guidelines (NHS CRD 2009). No constraints were set on the economic study type and limited criteria on the population of interest. The study parameters were therefore wide in scope, which successfully facilitated a search of literature to inform the knowledge base of cost‐effectiveness of A/MBIs. Consistent with previous reviews that have evaluated the efficacy of A/MBIs, the studies included in the current review focused on a range of conditions (Gotink *et al*., 2015; Khoury *et al*., 2013) and interventions (Cavanagh *et al*., 2014; Veehof *et al*., 2016; Vøllestad *et al*., 2012). Although this is in keeping with the transdiagnostic aspirations and credentials of A/MBIs, the heterogeneity of included populations, interventions, and economic evaluation study types limits the extent to which firm conclusions can be made in relation to different clinical conditions. An additional potential source of heterogeneity was the inclusion of economic evaluations assessing diverse forms of A/MBIs including MBCT, ACT, and DBT. This approach is, however, consistent with other systematic reviews and meta‐analyses of A/MBIs that have also included studies evaluating MBCT, ACT, and DBT (Fuchs, Lee, Roemer, & Orsillo, 2013; Godfrey, Gallo, & Afari, 2015; Wanden‐Berghe, Sanz‐Valero, & Wanden‐Berghe, 2010). Although these approaches have different theoretical bases, each advocates for mindfulness practices as key components to the interventions. It is important to note that DBT was only assessed in relation to EUPD. As the results are presented separately by mental health condition, the health economic evaluation for DBT can be easily disaggregated from the analyses relating to other forms of A/MBIs.

### Future research and policy implications

This is the first review to systematically synthesize research evidence relating to the cost‐effectiveness of A/MBIs. As such, the review makes an important contribution to guiding future research and decision‐making relating to service design and delivery. Firstly, whilst there were a number of technical issues with the three studies examining the cost‐effectiveness of MBCT on depression, there was a relatively high level of internal and external validity across these studies. NICE (2009) recommended MBCT as a treatment for people who are currently well but had experienced three or more previous depressive episodes. This decision was made based on one cost‐effectiveness analysis (Kuyken *et al*., 2008), which produced an inconclusive result. This has been superseded by the study of Kuyken, Hayes, Barrett, Byng, Dalgleish, Kessler, Lewis, Watkins, Brejcha, *et al*. (2015a), which found the intervention not to be cost‐effective. Given the relatively poor implementation of MBCT for depression (Crane & Kuyken, 2013), the findings of the review provide information that may be relevant to decision‐makers responsible for implementation of this NICE guideline. Two recent reviews of MBIs have highlighted that there is a need to better monitor the quantity and quality of between‐session mindfulness practice, which is purported to be an important process of change (Lloyd, White, Eames, & Crane, 2017; Parsons, Crane, Parsons, Fjorback, & Kuyken, 2017). Moving forward, it may be that if greater standardization is brought to the quality and quantity of the practice, then the effectiveness and cost‐effectiveness of A/MBIs may be increased.

### Conclusion

This systematic review provides an evaluation of the current evidence base on the health economic impact of A/MBIs for a range of mental health disorders. The available economic evaluations varied in terms of study population, intervention type, and the economic evaluation methods. The fact that a range of A/MBIs across different types of mental health disorders has been evaluated from a health economic perspective is positive, especially in the light of the limited understanding of what type of economic evidence existed before the review. The included economic evidence presented limitations, and therefore, the results should be interpreted with some caution. The results of the included economic evaluations varied with one study suggesting A/MBIs not to be cost‐effective whilst other studies were inconclusive. Three out of seven full economic evaluations suggest A/MBIs to be cost‐effective, whilst the three partial economic evaluations considered A/MBIs to be cost‐saving versus a range of comparator interventions. More research is needed before firm conclusions can be reached on the cost‐effectiveness of A/MBIs for mental health conditions.

## Funding

None of the authors had any direct or indirect funding in support of this study.

## Ethical approval

This article does not contain any studies with human participants performed by any of the authors.

## Supporting information


**Appendix S1.** Database: Ovid MEDLINE(R) <1946 to Present> 22nd November 2017.

**Appendix S2.** Methodological quality assessment using the Consensus on Health Economic Criteria (CHEC) checklist.
