# Supplementary material for: Are acceptance and mindfulness‐based interventions ‘value for money’? Evidence from a systematic literature review
Source: Br J Clin Psychol. 2018 Nov 29;58(2):187–210. doi: 10.1111/bjc.12208 (PMC6588093; doi:10.1111/bjc.12208)
Supplement: Supplementary file 1 — Appendix S1. Database: Ovid MEDLINE(R) <1946 to Present> 22nd November 2017. [file BJC-58-187-s001.docx]

**Supplementary Material 1**

Database: Ovid MEDLINE(R) <1946 to Present> **22^nd^ November 2017**

Search strategy:

--------------------------------------------------------------------------------

1 exp Mindfulness/

2 exp Meditation/

3 (mindful* or meditat* or MBSR* or MBCT).ti,ab.

4 (accept* adj4 commit* adj4 therap*).ti,ab.

5 (mindful* adj4 relapse* adj4 prevent*).ti,ab.

6 (Mindful* adj4 Stress* adj4 Reduc*).ti,ab.

7 (mindful* adj4 Cognit* adj4 Therap*).ti,ab.

8 (Dialect* adj4 Behavio?r* Therap*).ti,ab.

9 MBRP*.ti,ab.

10 DBT*.ti,ab.

11 "breathing technique*".ti,ab.

12 "breathing exercise*".ti,ab.

13 or/1-12

14 exp mental health/ or exp mental disorders/ or exp epilepsy/

15 (anxiet* or depress* or border* personalit* disorder* or mental health or mental disord* or Bipolar* or Neurocognit* or Neurodevelop* or Neurotic* or Paraphil* or Personalit* Disord* or Schizophren* or Psychotic* Disorder* or Sex* Dysfunct* or psychologic* or Sleep Disorder* or Somatoform* or Substanc* Relat* Disorder* or Trauma* or stress* or self* harm* or Season* affect* disord* or Post traumat* stress* disorder* or epilep*).ti,ab.

16 14 or 15

17 Economics/

18 "costs and cost analysis"/

19 Cost allocation/

20 Cost-benefit analysis/

21 Cost control/

22 Cost savings/

23 Cost of illness/

24 Cost sharing/

25 "deductibles and coinsurance"/

26 Medical savings accounts/

27 Health care costs/

28 Direct service costs/

29 Drug costs/

30 Employer health costs/

31 Hospital costs/

32 Health expenditures/

33 Capital expenditures/

34 Value of life/

35 exp economics, hospital/

36 exp economics, medical/

37 Economics, nursing/

38 Economics, pharmaceutical/

39 exp "fees and charges"/

40 Economics, Dental/

41 exp budgets/

42 (low adj cost).mp.

43 (high adj cost).mp.

44 (health?care adj cost$).mp.

45 (fiscal or funding or financial or finance).tw.

46 (cost adj estimate$).mp.

47 (cost adj variable).mp.

48 (unit adj cost$).mp.

49 (economic$ or cost or costs or costly or costing or price or prices or pricing or pharmacoeconomic$).ti,ab.

50 or/17-49

51 13 and 16 and 50

52 remove duplicates from 51
