# Supplementary material for: Are acceptance and mindfulness‐based interventions ‘value for money’? Evidence from a systematic literature review
Source: Br J Clin Psychol. 2018 Nov 29;58(2):187–210. doi: 10.1111/bjc.12208 (PMC6588093; doi:10.1111/bjc.12208)
Supplement: Supplementary file 2 — Appendix S2. Methodological quality assessment using the Consensus on Health Economic Criteria (CHEC) checklist. [file BJC-58-187-s002.docx]

**Supplementary Material 2**

Methodological quality assessment using the Consensus on Health Economic Criteria (CHEC) checklist

|  | Amner (2012) | Finnes et al. (2017) | Knight et al. (2015) | Kuyken et al. (2008) | Kuyken et al. (2015) | Pasieczny and Connor (2011) | Priebe et al. (2012) | Shawyer et al. (2016) | van Ravesteijn et al. (2013) | Wagner et al. (2014) |
| --- | --- | --- | --- | --- | --- | --- | --- | --- | --- | --- |
| 1. Is the study population clearly described? | Yes | Yes | Yes | Yes | Yes | Yes | Yes | Yes | Yes | Yes |
| 2. Are competing alternatives clearly described? | Yes | Yes | Yes | Yes | Yes | Yes | Yes | Yes | Yes | Yes |
| 3. Is a well-defined research question posed in answerable form? | Yes | Yes | Yes | Yes | Yes | Yes | Yes | Yes | Yes | Yes |
| 4. Is the economic study design appropriate to the stated objective? | Yes | Yes | Yes | Yes | Yes | No | Yes | Yes | Yes | Yes |
| 5. Is the chosen time horizon appropriate to include relevant costs and consequences? | Yes | Yes | Yes | Yes | Yes | Yes | Yes | Yes | Yes | Yes |
| 6. Is the actual perspective chosen appropriate? | Yes | Yes | Yes | Yes | Yes | Yes | Yes | Yes | Yes | Yes |
| 7. Are all important and relevant costs for each alternative identified? | No | Yes | Unclear | Yes | Yes | Yes | Yes | Yes | Yes | Yes |
| 8. Are all costs measured appropriately in physical units? | Yes | Yes | Unclear | Yes | Yes | Yes | Yes | Yes | Yes | Yes |
| 9. Are costs valued appropriately? | Yes | Yes | Unclear | Yes | Yes | Yes | Yes | Yes | Yes | Yes |
| 10. Are all important and relevant outcomes for each alternative identified? | n/a | Yes | n/a | Yes | Yes | Yes | Yes | Yes | Yes | n/a |
| 11. Are all outcomes measured appropriately? | n/a | Yes | n/a | Yes | Yes | Yes | Yes | Yes | Yes | n/a |
| 12. Are outcomes valued appropriately? | n/a | Yes | n/a | Yes | Yes | Yes | Yes | Yes | Yes | n/a |
| 13. Is an incremental analysis of costs and outcomes performed? | n/a | Yes | n/a | Yes | Yes | No | Yes | Yes | Yes | No |
| 14. Are all future costs and outcomes discounted appropriately? | No | n/a | No | No | Yes | n/a | n/a | No | n/a | No |
| 15. Are all important variables, whose values are uncertain, appropriately subjected to sensitivity analysis? | Yes | Yes | No | No | Yes | No | No | Yes | Yes | Yes |
| 16. Do the conclusions follow from the data reported? | Yes | Yes | Yes | Yes | Yes | No | Yes | Yes | Yes | Yes |
| 17. Does the study discuss the generalizability of the results to other settings and patient/client groups? | Yes | Yes | No | Yes | Yes | Yes | Yes | Yes | Yes | Yes |
| 18. Does the article indicate that there is no potential conflict of interest of study researcher(s) and funder(s)? | No | Yes | Yes | Yes | Yes | No | Yes | Yes | Yes | No |
| 19. Are ethical and distributional issues discussed appropriately? | No | No | No | No | No | No | No | No | No | No |
